# Cost-Effectiveness of PARP Inhibitors for Patients with BRCA1/2-Positive Metastatic Castration-Resistant Prostate Cancer—The Canadian Perspective

**DOI:** 10.3390/cancers17010040

**Published:** 2024-12-26

**Authors:** Ivan Yanev, Armen G. Aprikian, Brendan L. Raizenne, Alice Dragomir

**Affiliations:** 1 Centre for Outcomes Research and Evaluation, Research Institute of McGill University Health Centre, Montreal, QC H4A 3J1, Canada; yanev.ivan@mail.mcgill.ca; 2 Experimental Surgery, McGill University, Montreal, QC H3A 0G4, Canada; 3 Division of Urology, Department of Surgery, McGill University, Montreal, QC H3A 0G4, Canada; 4 Division of Urology, Centre Hospitalier de l’Université de Montréal, 900 St. Denis, Montreal, QC H2X 0A9, Canada; 5 Faculty of Pharmacy, University of Montreal, 2940 Chem. de Polytechnique, Montreal, QC H3T 1J4, Canada

**Keywords:** prostate cancer, mCRPC, PARP inhibitors, cost-effectiveness, partitioned survival model, healthcare system perspective, olaparib, rucaparib, talazoparib

## Abstract

Background/Objectives: Through phase III clinical trials, PARP inhibitors have demonstrated outcome improvements in mCRPC patients with alterations in BRCA1/2 genes who have progressed on a second-generation androgen receptor pathway inhibitor (ARPI). While improving outcomes, PARP inhibitors contribute to the ever-growing economic burden of PCa. The objective of this project is to evaluate the cost-effectiveness of PARP inhibitors (olaparib, rucaparib, or talazoparib) versus the SOC (docetaxel or androgen receptor pathway inhibitors (ARPI)) for previously progressed mCRPC patients with BRCA1/2 mutations from the Canadian healthcare system perspective. Methods: Partitioned survival models were created to represent mCRPC disease after progression until death. Survival inputs for BRCA1/2-mutated patients were extracted from the PROfound, TRITON3, and TALAPRO-1 clinical trials, while Canadian-specific costs are presented in 2023 dollars. Upon progression, patients were treated with chemotherapy. The considered time horizon was 5 years and outcomes were discounted at 1.5% per year. Results: PARP inhibitors provide an additional survival of 0.19 quality-adjusted life years (QALY) when compared to the current standard of care, with additional costs of CAD 101,679 resulting in an incremental cost-utility ratio (ICUR) of CAD 565,383/QALY. The results were most sensitive to PARP inhibitors’ acquisition costs and health-state utilities. PARP inhibitors required price reductions of up to 83% to meet the CAD 50,000/QALY willingness-to-pay threshold (WTP). Conclusions: While providing survival benefits to previously progressed mCRPC patients presenting deleterious BRCA1/2 gene mutations, PARP inhibitors are not cost-effective and require major price reductions to reach local WTP thresholds.

## Simple Summary

While considering previously progressed metastatic castration-resistant prostate cancer (mCRPC) patients presenting deleterious BRCA1/2 mutations, poly(adenosine diphosphate–ribose) polymerase (PARP) inhibitors provide survival benefits over the current standard of care (SOC). PARP inhibitors are, however, associated with additional costs and their use is constrained by access to genetic testing, potentially placing an economic strain on the healthcare system. This paper aims to assess the cost-effectiveness of PARP inhibitors as a therapeutic class and not only as a single agent, considering approved and soon-to-be approved therapies. This flexible approach considers multiple treatment options comparing the SOC and PARP inhibitors to represent more accurately the clinical management of mCRPC and the potential impact of PARP inhibitors on the treatment landscape. Results from the Canadian healthcare system perspective suggest that PARP inhibitors are not cost-effective. This research impacts policymakers, guiding future reimbursement negotiations and future health economic research in the field.

## 1. Introduction

### 1.1. Background

Metastatic castration-resistant prostate cancer (mCRPC) is the terminal stage of prostate cancer (PCa) and is responsible for most of the disease-specific mortality with an estimated median overall survival (OS) of 3 to 4 years [1,2]. In the last two decades, the treatment landscape for mCRPC has evolved substantially and treatments such as docetaxel, abiraterone acetate, and enzalutamide have demonstrated a clinical improvement in survival in randomized clinical trials (RCTs) and have become the current standard of care (SOC) for treating mCRPC [3,4,5,6,7]. Docetaxel is an IV-administered chemotherapy that first demonstrated effectiveness in this setting and became the treatment of choice. Consequently, abiraterone and enzalutamide, oral androgen receptor pathway inhibitors (ARPIs), demonstrated their effectiveness in docetaxel-progressed patients and have since then been widely accepted as a first-line treatment for mCRPC. While enzalutamide appears to be favored by urologists due to its lesser cardiovascular toxicity and easier patient surveillance, abiraterone generic options have entered the market by presenting a more economical alternative [8].

### 1.2. Mechanism of Action

More recently, PARP inhibitors have emerged as a promising class of therapeutic agents in the realm of cancer treatments for patients suffering from breast and ovarian cancer and are demonstrating valuable benefits for prostate cancer patients [9]. PARP inhibitors disrupt the single-strand break (SSB) DNA repair process by binding PARP1, preventing the recruitment of target proteins, facilitating the restructuring of chromatin around damaged DNA, and preventing bound PARP1 to release from DNA [10,11]. The hypothesis behind the mechanism of action of PARP inhibitors is that patients presenting homologous recombination repair gene (HRR) mutations that impair double-strand break (DSB) DNA repair, such as BRCA 1/2 gene mutations, will rely on a PARP-mediated SSB DNA repair mechanism to correct DNA damage [12]. Therefore, inhibiting PARP enzymes will lead to the cell death of cancer cells that accumulate greater DNA damage than normal cells. Additionally, there is an unmet need reported throughout the scientific literature, as BRCA1/2 mCRPC patients experience worse outcomes than unselected patients when treated with the current SOC [13].

### 1.3. Clinical Data

In recent results emerging from phase III RCTs, olaparib, rucaparib, and talazoparib, which inhibit PARP, have demonstrated outcome improvements in mCRPC patients with alterations in BRCA1/2, which have progressed on ARPI [14,15,16]. The PROfound trial demonstrated that olaparib was associated with a longer progression-free (PFS) survival and better measures of response than either enzalutamide or abiraterone [15]. This resulted in a Health Canada approval of olaparib for the treatment of adult patients with deleterious or suspected deleterious germline and/or somatic BRCA- or ATM-mutated mCRPC [17] and is being reimbursed at the provincial level [18,19,20]. The TRITON 3 trial reported similar results, demonstrating a significantly longer imaging-based PFS in mCRPC patients that presented a BRCA mutation when compared to the SOC (docetaxel or ARPI) [16]. TALAPRO-1 is a single-arm trial that analyzed the benefits of talazoparib in mCRPC patients with different HRR mutations and demonstrated that patients expressing deleterious BRCA1/2 mutations have a more favorable response and better survival than any other HRR mutations. Currently, rucaparib and talazoparib are not yet approved by Health Canada for the treatment of mCRPC.

### 1.4. Rationale

While improving patient outcomes, PARP inhibitors are associated with additional costs that are significantly higher than generic options (e.g., docetaxel or abiraterone) or even non-generic options (enzalutamide) and contribute to the ever-growing economic burden of PCa. In Canada, genetic testing for BRCA1/2 is publicly covered and is required to gain access to treatment with PARP inhibitors. As these PARP inhibitors are soon to enter the treatment landscape for mCRPC, their affordability and cost-effectiveness need to be assessed as a therapeutic class, to estimate their economic impact on the healthcare system and guide health policy decision making to better allocate health resources. While there is no explicit willingness-to-pay (WTP) threshold for cost-effectiveness in Canada, the common literature refers to CAD 50,000 and CAD 100,000 per quality-adjusted life year (QALY) [21]. The aim of this research is to assess the cost associated with PARP inhibitors that will be inflicted on the Canadian Healthcare system (acquisition- and treatment-related costs) in comparison to their expected effectiveness, benchmarking them against the current SOC through sequential analysis. These results will be benchmarked against the commonly referred WTP thresholds.

### 1.5. Objectives

The primary objective of this study is to evaluate the cost-effectiveness of PARP inhibitors (olaparib, rucaparib, or talazoparib) versus the current SOC (docetaxel or ARPI) for mCRPC patients with BRCA1/2 mutations from a Canadian healthcare system perspective. Furthermore, treatment acquisition costs tend to have an important effect on the economic impact and play a significant role in negotiations for public reimbursement. As a secondary objective, this project aims to determine a suitable price for PARP inhibitors, which would satisfy local cost-effectiveness WTP requirements through a price threshold analysis. These projections will provide a guide for a pricing strategy of PARP inhibitors in the Canadian healthcare system.

## 2. Materials and Methods

### 2.1. Target Population

The target population was mCRPC-confirmed male patients in Canada with a BRCA1/2 mutation who have progressed on ARPI as described in the PROFound, TRITON 3, and TALAPRO-1 trials [14,15,16]. Patients presented either a germline or somatic BRCA1/2 mutation, confirmed by genetic testing.

### 2.2. Therapeutic Options and Comparators

The studied therapeutic PARP inhibitor options are olaparib 300 mg orally administered twice daily, rucaparib 600 mg orally administered twice daily, or talazoparib 1 mg orally administered daily. In the base case model, the PARP inhibitor strategy is defined by equal proportions of olaparib, rucaparib, and talazoparib. In the base case analysis, the SOC therapeutic options are split 2:1 between chemotherapy (docetaxel 75 mg/m^2^ every 3 weeks for 6 cycles) and ARPI (abiraterone 1000 mg + prednisone 5 mg daily or enzalutamide 160 mg daily). The abiraterone and enzalutamide split for the ARPI arm was 33.6% to 66.4%, respectively, based on a populational study by George et al., 2020 [22]. Different proportions of therapy usage for the SOC were explored in a scenario analysis. The base case analysis aims to compare the PARP inhibitor therapeutic options and the current standard of care. These results are further stratified by specific therapies to demonstrate the impact on the incremental cost-utility ratio (ICUR) and provide more granular results.

### 2.3. Model Design and Parameters

#### 2.3.1. Setting and Location

**Figure 1:**
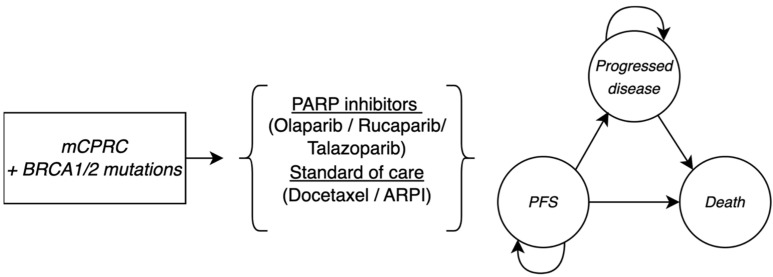
Partitioned survival model structure.

We designed a three-health-state partitioned survival model (Figure 1) in TreeAge Pro© Healthcare (version 2024 (x86_64)) to reproduce the secondary care outpatient setting, while incorporating Canadian-specific inputs and using the healthcare system perspective. This model represents patients’ pathways until death. The progression-free survival (PFS) state represents time from the treatment initiation of PARP inhibitors of the SOC of the target population. Possible transitions are described by arrows leading to the “Progressed disease” health state, where patients are introduced to a subsequent line of treatment (chemotherapy) due to the radiographic progression of their disease. Once patients progress, they can no longer return to the PFS state. Death can occur from all the health states and is designed to reproduce overall survival (OS) observed in clinical trials. Transitions are further explained in the Clinical Inputs for Efficacy section. Curved arrows indicate patients remaining in their current health state.

#### 2.3.2. Time Horizon

A patient lifetime horizon was chosen for this analysis. We monitored survival to ensure that the majority of the simulated patients have reached the terminal health state by the end of the modelled time of 5 years. Given the advanced stage of the patients, this time horizon is appropriate and was in line with the literature [23]. A 10-year time horizon was explored in a scenario analysis. Outcomes are discounted by 1.5% annual rate as per the Canadian Drug Agency’s (CDA’s) most recent guidelines [24].

### 2.4. Clinical Inputs for Efficacy

#### Outcomes (Selection, Measurement, and Valuation)

We used radiographic progression-free survival (rPFS) curves reported by clinical trials to model patient progression and transition to a subsequent line of treatment. We modelled patient survival through published OS curves, and extracted rPFS and OS of the BRCA/12 subpopulations from the PROfound, TRITON 3, and TALAPRO-1 trial publications with the DigitizeIt© (version 2.4.2) software [14,15,16]. Population characteristics from trials are presented in Table A1. We reconstructed patient-level time to event data with the protocol of Guyot et al. and parametrized the survival data in R© (version 4.3.1) [25]. This protocol was used as it effectively allows recreating patient-level time to event data, taking into consideration censoring observed in clinical trials, while being widely validated by the scientific community. Results from the reconstructed curves were benchmarked by comparing captured events and by visual inspection against published survival curves. We tested Log-Logistic, Exponential, Weibull, Generalized Gamma, Gamma, Log-Normal, and Gompertz parametric models to parametrize extracted data. Model selection was performed based on the lower Akaike information criterion (AIC) of the tested models and visual confirmation was used to verify that extrapolated data were supported by observed data. Model internal validity was benchmarked against survival curves observed in clinical trials. Furthermore, we validated that PFS and OS curves did not present crossings and respected the requirements for partitioned survival analyses. We extracted health-state utility values for PFS and progressed disease (PD) from Xu et al. representing health quality data from the PROfound trial [15,23]. We integrated a Canadian-specific valuation of the health-state utilities by anchoring it to the general Canadian population health utility values of the target population [26,27]. Refer to Table 1 for survival and utility inputs.

### 2.5. Costs

#### 2.5.1. Currency Price Date and Conversion

Costs used in the model are updated to 2023 CAD by using the Canadian healthcare-specific consumer price index method based on data available from Statistics Canada [29] (Table 2).

#### 2.5.2. Treatment-Specific Costs

We calculated treatment-specific costs taking into consideration the acquisition and administration cost of treatments that we extracted from the RAMQ list of medications or the MUHC internal price list when costs were not publicly available. For chemotherapies, we considered premedication, medication, transfusion solutions, nurse time, and medical oncologist visits.

#### 2.5.3. Health State-Specific Costs and Genetic Testing

We calculated health state-specific costs based on follow-up care, physician routine visits, medical imaging, and laboratory tests needed to monitor patients within the given health state. Costs related to laboratory tests, including the BRCA1/2 genetic mutation testing, and imaging procedures were extracted from the Ministry of Health and Social Services (MHSS) costing manual [30], while costs related to medical acts were extracted from the “Manuel de la rémunération à l’act des Médecins spécialistes de la RAMQ” [31].

#### 2.5.4. Adverse Events Costs

We extracted costs associated with adverse events (AEs) from the Ontario Case Costing Initiative [32]. We considered AEs to be treated in the outpatient setting, except for febrile neutropenia, which was considered to be treated in the inpatient setting as it requires hospitalization.

**Table 2:** Costs in Canadian dollars.

Parameter	Mean	Range	Source
PARP inhibitors (per cycle)	CAD 7907	CAD 7116–CAD 8698	RAMQ medication list [18]
Enzalutamide (per cycle)	CAD 3401	CAD 3061–CAD 3742	RAMQ medication list [18]
Abiraterone + Prednisone (per cycle)	CAD 919	CAD 827–CAD 1011	RAMQ medication list [18]
Docetaxel (average per cycle) including premedication and additional fees	CAD 192	CAD 173–CAD 211	MUHC internal prices
Cabazitaxel (average per cycle) including premedication and additional fees	CAD 2405	CAD 2164–CAD 2645	MUHC internal prices
Laboratory testing (per cycle)	CAD 16	CAD 12–CAD 20	MHSS price list [30]
Bone imaging + CT (per cycle)	CAD 229	CAD 172–CAD 287	MHSS price list [30]
Palliative care per cycle	CAD 1784	CAD 1338–CAD 2231	de Oliveira et al., 2016 [33]
BRCA genetic testing	CAD 843	CAD 633–CAD 1054	MHSS price list [30]

### 2.6. Modelling Assumptions

This model recreates patients’ pathways from progressive mCRPC until death. After radiographic progression, patients were treated with chemotherapy; six (6) cycles of docetaxel or cabazitaxel if docetaxel was the initial treatment. As rucaparib and talazoparib are not currently being commercialized in Canada, we assumed their acquisition costs to be on par with olaparib, as it is for other treatments for advanced prostate cancer within the same class (i.e., enzalutamide, apalutamide, darolutamide, and abiraterone before the arrival of generic versions).

### 2.7. Sensitivity Analysis

We conducted deterministic and probabilistic sensitivity analysis where we varied drug acquisition costs by +/−10%, health state-specific costs, and AE costs by +/−25% to account for uncertainty and potential patient heterogeneity. In the probabilistic sensitivity analysis, the model was run as a Monte-Carlo simulation with 10,000 samples.

### 2.8. Price Threshold Analysis

We conducted price threshold analysis through a built-in function in TreeAge Pro© to determine the cost of PARP inhibitors and the potential price reduction needed to reach different WTPs for cost-effectiveness, ranging from CAD 50,000 to CAD 150,000 per QALY.

### 2.9. Scenario Analysis

We conducted a scenario analysis exploring different distributions of ARPI usages to explore how the cost of ARPI will affect the cost-effectiveness results. We explored 100%, 75%, 50%, and 0% of the population on abiraterone acetate, while the remaining portion was on enzalutamide. As the ARPI strategy was presented as a hole in TRITON 3, and no stratification based on individual treatment, we only adapted the costing associated with the different scenarios. We conducted a scenario analysis exploring a 10-year time horizon. To conduct this scenario analysis, we employed a parametric model to represent PFS data for rucaparib following a Gamma distribution. This model had a higher AIC than the log-logistic model by 3.99 points but prevented survival curve crossing when extrapolating the longer time horizon.

## 3. Results

### 3.1. Summary of Results and Study Parameters

**Figure A1:**
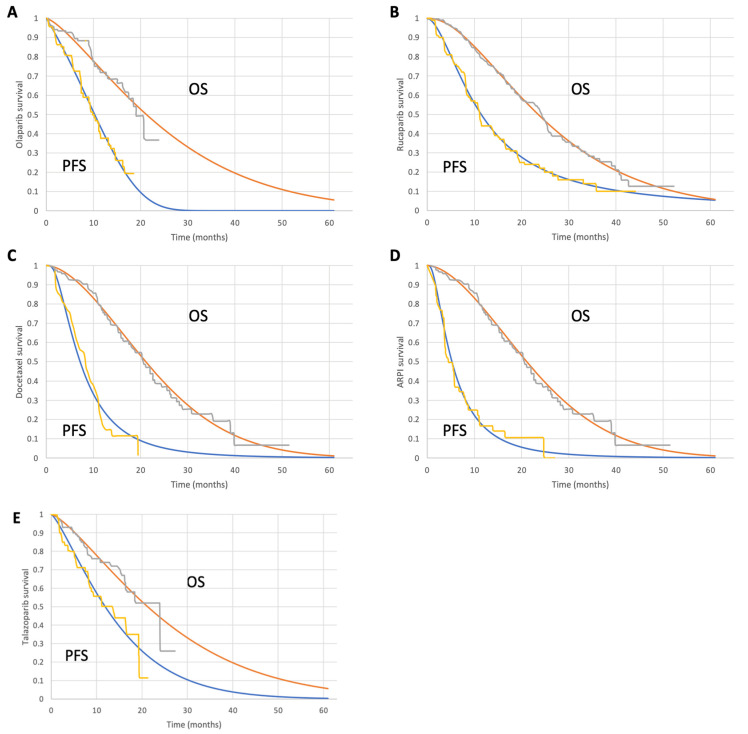
Validation of the best-fitted parametric curves on reconstructed Kaplan–Meier curves from PROfound, TRITON3, and TALAPRO-1. Panel (**A**), olaparib survival data; Panel (**B**), rucaparib survival data; Panel (**C**), docetaxel survival data; Panel (**D**), ARPI survival data; Panel (**E**), Talazoparib survival data. Digitized overall survival (OS) curve from clinical trials in grey; digitized progression-free survival (PFS) curve from clinical trials in yellow; modelled PFS curve in blue; modelled OS curve in orange. (ARPI, androgen receptor pathway inhibitors).

We validated the modelled OS and PFS parametric curves, benchmarking them against patient survival reported in clinical trials to demonstrate internal model validity and present survival extrapolation beyond the time horizon studied in clinical trials (Figure A1). Within this validation, we observed that only 2% of rucaparib’s modelled effectiveness emerged from the extrapolated survival while 23% and 20% of the respective olaparib and talazoparib modelled effectiveness were associated with the extrapolated survival, as PROfound and TALAPRO-1 had shorter reported follow-up periods.

### 3.2. Effectiveness, Costs, and Cost-Utility Results

In terms of discounted effectiveness for previously progressed BRCA1/2 mCRPC patients, PARP inhibitors were associated with 2.07 life years (LY) or 1.02 QALY while the SOC provided 1.88 LY or 0.84 QALY. While being more effective, PARP inhibitors were associated with higher costs, CAD 101,769 more than the SOC (CAD 60,987), which resulted in an ICUR of CAD 565,383/QALY in the base case analysis (Table 3).

**Figure A2:**
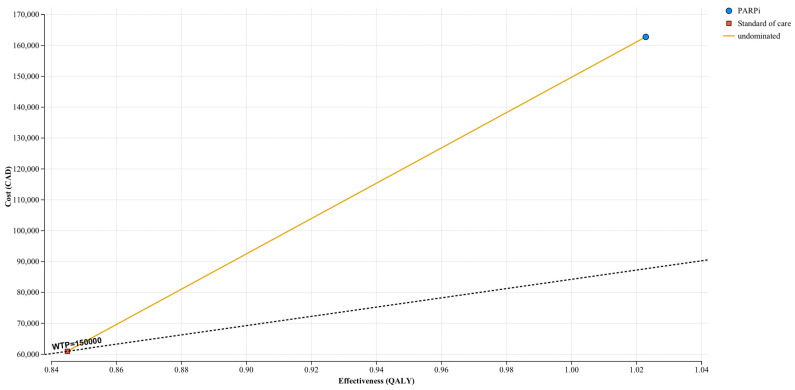
Cost-effectiveness frontier.

The cost-effectiveness frontier of this analysis demonstrates that PARP inhibitors are not cost-effective at their current price. Considering the modelled PARP inhibitors’ effectiveness, the total strategy costs need to be closer to CAD 90,000 to reach a WTP threshold of CAD 150,000/QALY (Figure A2).

When decomposing these results by therapy, we observed that the ARPI strategy was absolutely dominated by docetaxel as it presented a lower effectiveness at a higher cost, primarily led by the high acquisition costs of enzalutamide (Table A2). When compared to docetaxel, olaparib is associated with an ICUR of CAD 858,456/QALY and talazoparib with an ICUR of CAD 671,244/QALY and were extendedly dominated (dominated by the combination of treatments, docetaxel and rucaparib in this instance). Rucaparib was the only therapy that was undominated and resulted in an ICUR of CAD 494,254/QALY. All PARP inhibitor options resulted in ICURs exceeding local WTP thresholds.

When conducting a pairwise comparison between olaparib and the other PARP inhibitors, we observed ICURs of CAD 411,580/QALY and CAD 292,626/QALY vs. talazoparib and rucaparib, respectively, mainly driven by finite incremental effectiveness. The pairwise comparison between talazoparib and rucaparib yields an ICUR of CAD 207,145/QALY. While these therapies present life-prolonging benefits, their incremental effectiveness between therapeutic options is exiguous especially when expressed in QALY, as these survival benefits are associated with poor quality of life. Furthermore, costs are explosively accumulated over these short periods of added survival, which explains the high ICURs between therapeutic options.

### 3.3. Sensitivity and Price Threshold Analysis

**Figure 2:**
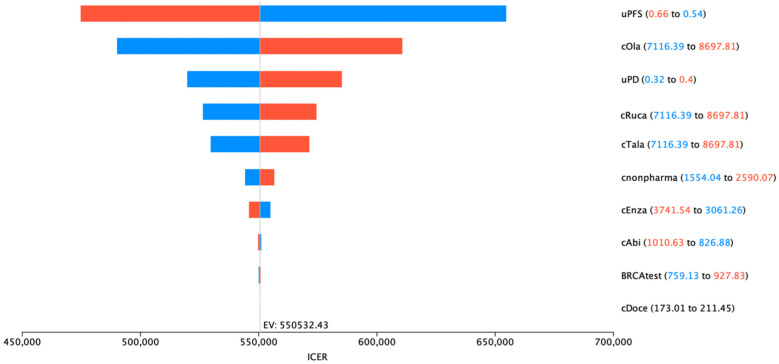
Tornado diagram of deterministic sensitivity analysis. Parameter increase is expressed in red, while blue denotes parameter decrease. Expected value (EV) indicates base case ICER results.

**Figure 3:**
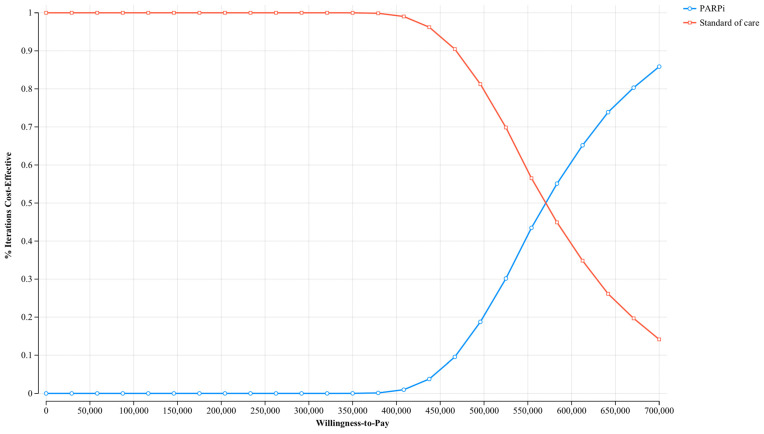
Cost-effectiveness acceptability curve (willingness-to-pay displayed in Canadian dollars).

**Figure A3:**
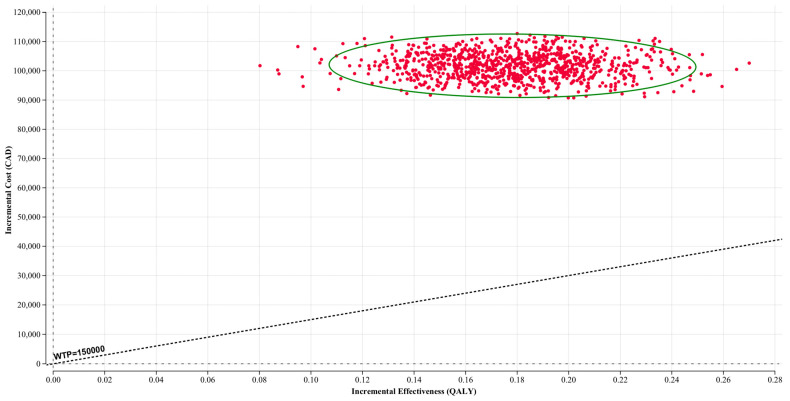
Incremental cost-effectiveness scatterplot, PARP inhibitors vs. standard of care. Green ellipsis represents 95% confidence interval.

The results from the deterministic sensitivity analysis highlighted PFS utility, progressed-disease (PD) utility, and the cost of PARP inhibitors as parameters with the greatest impact on the ICUR results (Figure 2). However, no single parameter variation was able to decrease the ICURs under the common WTP thresholds. The results from the probabilistic sensitivity analysis demonstrated that PARP inhibitors are associated with higher costs than the SOC but will consistently yield a higher treatment effectiveness (Figure A3). While all the samples are contained in the first quadrant of the cost-effectiveness plain, indicating that PARP inhibitors are more effective than the SOC, these samples are also above the CAD 150,000/QALY WTP threshold line, indicating that they are not cost-effective. The acceptability curve that was generated through this sensitivity analysis demonstrated that due to high ICURs, the current SOC presents the most cost-effective treatment option, unless the WTP thresholds exceed CAD 570,000/QALY (Figure 3). At the predefined WTP thresholds of CAD 50,000, CAD 100,000, and CAD 150,000/QALY, PARP inhibitors required a reduction in the list price of 83%, 75%, and 66%, respectively, to reach these thresholds of cost-effectiveness (Table 4).

### 3.4. Scenario Analysis Results

Scenario analysis exploring different proportions of ARPI usage demonstrated that if only abiraterone is considered at its generic price, the APRI option is no longer being dominated by docetaxel and will yield an ICUR of CAD 214,350/QALY (Table 5). However, the ARPI option becomes dominated (more expensive while less effective) as soon as 25% of the ARPI patients are administered enzalutamide. We explored a scenario analysis, where chemotherapy and ARPI were administered at equal proportions in the SOC (1:1). This scenario reported an ICUR of CAD 549,467/QALY when comparing the PARP inhibitors to the standard of care, underlining the fact that ARPI is not a cost-effective option when the current list prices of enzalutamide are taken into consideration.

The results from the exploratory 10-year time horizon scenario analysis confirmed the base case results. In fact, extending the time horizon yields an ICUR of CAD 526,426/QALY where PARP inhibitors demonstrated an effectiveness of 1.04 QALY at a cost of CAD 161,315 and the SOC 0.85 QALY at a cost of CAD 61,294. These results underline the model robustness and demonstrate that the results are mature enough at 5 years, as the majority of the outcomes are already observed.

## 4. Discussion

### 4.1. Summary of Results

This analysis demonstrated that at Canadian WTP thresholds, PARP inhibitors are not a cost-effective option for treating previously progressed mCRPC patients that are expressing a deleterious BRCA1/2 mutation, albeit PARP inhibitors present an effective option that provides patients additional QALY as an alternative to chemotherapy. Rucaparib is associated with an ICUR of CAD 494,254/QALY, while olaparib and talazoparib were less cost-effective options. The ARPI treatment regimen was associated with higher costs while being less effective than docetaxel. However, the scenario analysis demonstrated that if generic prices are considered, ARPI could potentially be cost-effective and remain an option for patients that are unfit for chemotherapy. PARP inhibitors require 83% list price reductions to reach common Canadian cost-effectiveness thresholds.

### 4.2. Comparison to the Literature

As per our knowledge, this is the first analysis that compared a combined PARP inhibitors strategy of olaparib, rucaparib, and talazoparib to the SOC in the Canadian healthcare system. When comparing these results to the current literature, our results align with other cost-effectiveness studies. Xu et al. analyzed the cost-effectiveness of olaparib compared to ARPI from the US and Chinese perspectives through a Markov model [23]. Their findings report that olaparib is not cost-effective in China but could be cost-saving in the US, as ARPI prices in the US are relatively high. Our analysis differs as we complemented survival data with findings from the TRITON 3 trial and added docetaxel as a comparator and rucaparib and talazoparib as part of the PARP inhibitors options. We did not observe cost savings in the Canadian setting as abiraterone and docetaxel have generic options at significantly lower costs. Additionally, our findings are comparable to the CDA reimbursement recommendation of olaparib (Lynparza), where olaparib was associated with an ICUR of CAD 459,527/QALY when compared to docetaxel and required a 71% price reduction to reach the CAD 50,000/QALY WTP [34]. Our price threshold analysis and combined PARP inhibitors scenario analysis confirm these findings. Our methodology slightly differs from the CDA’s, as we were able to use mature data from the TRITON 3 trial where docetaxel was used as part of the control arm. With the suggested price reductions, PARP inhibitors could present an additional option for BRCA1/2-mutated mCRPC patients that could meet local cost-effectiveness thresholds. Ding et al. conducted a systematic literature review of the cost-effectiveness analyses of PARP inhibitors in different cancers [35]. Their findings suggest that in the treatment of breast cancer, PARP inhibitors are not cost-effective when compared to chemotherapy as they exceed WTP thresholds (WTP thresholds ranging up to USD 150,000 per QALY). Similar conclusions can be made in the treatment of pancreatic and ovarian cancer. Ding et al. confirmed our observations as they identified the acquisition costs of PARP inhibitors to be the limiting factor in their cost-effectiveness study.

Treatment availability could potentially be impacted by genetic testing. While these tests are publicly covered, their costs do not impact ICURs as shown in this analysis. The access to these tests can, however, be limited due to test unavailability in remote regions and time to obtain test results. The estimated number of patients per year and how patients have access to testing (geographical setting, testing criteria, test availability, etc.) might have an impact on access to treatment. The current Canadian Urological Association guidelines recommend that germline testing be performed in metastatic prostatic cancer patients and genomic profiling of the tumor in mCRPC patients should be performed while BRCA1/2 are among the targeted genes [36]. In their recent report, the CDA noted that the availability of these genetic testing and diagnosis varies across Canada and could be negatively impacted by the increased demand and affect timely access [37].

It is important to consider that the size of the patient population is restricted, as these are terminal patients that have not only progressed on first-line therapies for advanced disease but present a specific mutation. It is reported that only 4.4% of mCRPC present a germline BRCA1/2 mutation in Canada [36]. As administration is contingent on confirmation of the genetic mutation and access to companion diagnostics can be limited due to their availability, access to PARP inhibitors could potentially be decreased. This will result in a diminished number of treated patients per year and could decrease the budget impact on the healthcare system. A complete budget impact analysis should be conducted to assess how the Canadian healthcare system will be impacted by the addition of PARP inhibitors for the specific patient population. Talazoparib is currently being reviewed by Health Canada and is being evaluated by CADTH in combination with enzalutamide for the first-line treatment of mCRPC. A favorable funding recommendation by CADTH in the first-line setting could promote second-line usage, which sheds light on the importance of this study. Rucaparib is not currently being commercialized in Canada and does not have a notice of compliance from Health Canada, which can be an important hurdle to patient access. Going through the Canadian approval and reimbursement processes could potentially delay rucaparib’s access by several years, which significantly impairs patient benefits.

### 4.3. Limitations

Our study was faced with certain limitations. Firstly, the lack of stratification by therapy in the SOC arm of the TRITON 3 trial forced us to approximate that patients treated with ARPI and docetaxel will experience the same OS. While this is a limitation that will impact the granularity of the results, it will ultimately not affect the results for the SOC presented in this paper and it represents the most accurate available data.

Secondly, the TRITON 3 population could potentially be more advanced as 87% and 84% of the rucaparib and control patients, respectively, present metastasis to the bone, which could be higher than the PROfound patients [16]. Additionally, TRITON 3 allowed for the use of docetaxel to be part of the control arm, which was not the case for the PROfound trial, underlining the differences between these two trials. TALAPRO-1 was a single-arm, open-label trial, which could lead to potential bias. This more advanced population could be associated with less favorable survival and could potentially lead to an underestimation of the results associated with talazoparib. Nevertheless, these are the best currently available data for this study. Therefore, the results concerning talazoparib should be interpreted with caution.

Thirdly, as we used a parametric partitioned survival model to extrapolate survival data beyond the clinical trial observed period, the choice of a parametric model to extrapolate this survival incorporates a certain degree of bias [38]. Parametric curves reproduce survival observed in clinical trials (Figure A1). However, there is no certainty that these models can accurately extrapolate survival beyond the observed period. Conversely, this extrapolation could lead to an overestimation of the results, favoring PARP inhibitors. To mitigate this bias, we consulted with clinical experts to validate the extrapolated survival curves, reported the modelled survival curves against clinically reported survival (Figure A1), and reported the proportion of the modelled effectiveness that emerges from the extrapolated survival.

Partitioned survival analyses can be perceived as less flexible than Markov models and are critiqued for not incorporating time-varying effects. While this holds true, these limitations are minimal in the given terminal disease setting, where patients will not be receiving multiple subsequent lines of therapy. Additionally, the short time horizon controls and limits the time-varying effects while accurately modelling the entire patient survival. The terminal disease setting and the short time horizon negate the limitation associated with partitioned survival analysis while accurately reproducing survival observed in RCTs, justifying the choice of model.

The results from the exploratory 10-year time horizon confirmed our base case results and justify the appropriateness of a 5-year time horizon. While only exploratory, the 10-year time horizon extrapolates data well beyond the observed period in clinical trials and could potentially introduce bias, under or over-estimating survival results, as it is unlikely that advanced-disease PCa patients could reach the end of this time horizon. The results from this scenario should therefore be interpreted with caution.

As mentioned in the discussion section, the CUA guidelines recommend genetic testing of mCRPC patients, while the CDA states that the increased demand for testing could impact access to treatment. Unfortunately, it is too early to quantify how the scarcity of genetic testing will impact the cost-effectiveness of PARP inhibitors as data are still immature; this remains a limitation of this project.

Lastly, drug acquisition costs have an important impact on the modelled results. There are price disparities within different jurisdictions in Canada that can bias the generalizability of the results to the Canadian population. As the AE costs were extracted from the Ontario Case Costing Initiative, a certain bias was incorporated as these costs have not been updated since 2017 and are Ontario-specific, which might not be representative of the general Canadian landscape, as it is potentially more expensive to treat patients in the more remote rural areas of the country, albeit this source provided a robust costing evidence, which was indexed to the year of valuation to mitigate bias. Furthermore, as rucaparib and talazoparib are not commercialized in Canada, we hypothesized that they would have the same treatment acquisition costs as olaparib.

## 5. Conclusions

While providing survival benefit to mCRPC patients presenting alterations in BRCA1/2 genes, PARP inhibitors are not cost-effective when compared to the current standard of care. The economic burden of PARP inhibitors requires cost reduction scenarios to meet Canadian healthcare-perspective WTP thresholds, such as price reductions or volume-based rebates. As the landscape of PARP inhibitors is rapidly evolving with the arrival of novel treatments, including PARP inhibitors’ coadministration with APRI for the first-line treatment of mCRPC, further cost-effectiveness assessments of PARP inhibitors in the first-line treatment of mutated and unselected patients are needed to optimize resource allocation. While cost-effectiveness analyses could potentially justify the acquisition costs of PARP inhibitors, their affordability, or in other words, how publicly reimbursing these treatments will impact the healthcare budget needs to be assessed through budget impact analyses.

## Figures and Tables

**Table 1:** Survival and utility inputs.

Parameter	Shape/Mean Log	Scale/Rate/Sdlog/Sd	Distribution	Source
PFS curves				
Olaparib	0.086	0.0135	Gompertz	de Bono et al., 2020 [15]
Rucaparib	1.713	11.45	Log-Logistic	Fizazi et al., 2023 [16]
Docetaxel	1.9729	0.7646	Log-Normal	Fizazi et al., 2023 [16]
Talazoparib	1.276	15.852	Weibull	de Bono et al., 2021 [14]
ARPI	1.6579	0.8423	Log-Normal	Fizazi et al., 2023 [16]
OS curves				
Olaparib	1.610	24.93	Weibull	de Bono et al., 2020 [15]
Rucaparib	2.216	0.0805	Gamma	Fizazi et al., 2023 [16]
Docetaxel	1.766	25.974	Weibull	Fizazi et al., 2023 [16]
Talazoparib	1.347	27.885	Weibull	de Bono et al., 2021 [14]
ARPI	1.766	25.974	Weibull	Fizazi et al., 2023 [16]
Utilities				
PFS	0.60	0.0615	Beta	Robson et al., 2017 [28]
PD	0.36	0.022	Beta	Robson et al., 2017 [28]

**Table 3:** Cost-effectiveness results of PARP inhibitors vs. the standard of care.

Strategy	Cost	Incremental Cost	Effectiveness (LY)	Incremental Effectiveness (LY)	Effectiveness (QALY)	Incremental Effectiveness (QALY)	ICUR
Standard of care	CAD 60,987		1.88		0.84		
PARP inhibitors	CAD 162,755	CAD 101,769	2.07	0.19	1.02	0.18	CAD 565,383/ QALY

(ICUR, incremental cost-utility ratio; LY, life years; PARP, poly(adenosine diphosphate–ribose) polymerase; QALY, quality-adjusted life year.)

**Table 4:** Price threshold analysis and needed reduction to reach different willingness-to-pay thresholds.

WTP Threshold	Suggested PARP Inhibitors’ Price	Price Reduction from List Price
CAD 150,000	CAD 2655	66%
CAD 100,000	CAD 2000	75%
CAD 50,000	CAD 1344	83%

**Table 5:** Scenario analysis: proportion of patients on different ARPI strategies.

ARPI Proportion	Cost (CAD)	ICUR vs. Docetaxel (CAD/QALY)
Abi 100%/Enza 0%	CAD 54,064	CAD 214,350
Abi 75%/Enza 25%	CAD 58,713	Dominated
Abi 50%/Enza 50%	CAD 63,260	Dominated
Abi 0%/Enza 100%	CAD 72,421	Dominated

## Data Availability

Data are available upon request.

## Appendix A

**Table A1:** Population characteristics.

Trial	PROFound		TRITON 3		TALAPRO-1
	**Olaparib**	**ARPI**	**Rucaparib**	**ARPI + Doce**	**Talazoparib**
Median age at randomization	68	67	70	71	69
Race (% white)			74%	76%	87%
Gleason score 8+ (%)	67%	67%	64%	71%	64%
Bone metastasis	35% *	28% *	87%	84%	NA
Visceral metastasis	28%	39%	27%	34%	30%
ECOG PS					
0	52%	41%	49%	50%	39%
1	41%	55%	51%	50%	59%
2	7%	4%			10%
Previous taxane use					
Docetaxel	46%	39%	23%	21%	57%
Cabazitaxel	1%	0%			0%
Docetaxel + Cabazitaxel	18%	24%			43%
Previous NHT use					
Abiraterone	38%	35%	56%	59%	45%
Enzalutamide	42%	48%	44%	45%	33%
Abiraterone + Enzalutamide	20%	17%	0	0	20%

Bone metastasis only. (ARPI, androgen receptor pathway inhibitors; ECOG PS, Eastern Cooperative Oncology Group Performance Status; NA, not available; NHT, new hormonal therapy.) * Bone metastasis only.

**Table A2:** Cost-effectiveness results stratified by single therapeutic option.

Strategy	Cost	Incremental Cost	Effectiveness (LY)	Incremental Effectiveness (LY)	Effectiveness (QALY)	Incremental Effectiveness (QALY)	ICUR
Docetaxel	CAD 58,351		1.88		0.85		Reference
ARPI	CAD 66,256	CAD 7,905	1.88	0.00	0.83	−0.03	Abs. dominated
Olaparib	CAD 135,612	CAD 77,261	2.01	0.13	0.94	0.09	CAD 858,456/QALY (Ext. dominated)
Talazoparib	CAD 165,750	CAD 30,138	2.02	0.01	1.01	0.07	CAD 671,244/QALY (Ext. dominated)
Rucaparib	CAD 186,857	CAD 128,506	2.17	0.29	1.11	0.26	CAD 494,254/QALY
